# Effect of the essential oil of *Rosmarinus officinalis* (L.) on rooster sperm motility during 4°C short-term storage

**DOI:** 10.14202/vetworld.2018.590-597

**Published:** 2018-05-09

**Authors:** L. Touazi, B. Aberkane, Y. Bellik, N. Moula, M. Iguer-Ouada

**Affiliations:** 1 Ecole Nationale Supérieure Vétérinaire, Rue Issad Abbes, Oued Smar, Algiers, Algeria; 2 Associated Laboratory in Marine and Aquaculture Ecosystems, Faculty of Nature and Life Sciences, University of Bejaia. Algeria; 3 Department of Biology, Faculty of Nature and Life Sciences and Earth Sciences. University of Bouira, Algeria; 4 Department of Biology, Faculty of Nature and Life Sciences, University of El Bachir el Ibrahimi, Bordj Bou Arreridj, 34000, Algeria; 5 Department of Animal Productions, University of Liege, Faculty of Veterinary Medicine, 4000 Liege, Belgium

**Keywords:** antioxidant, avian semen, liquid storage, rosemary essential oil

## Abstract

**Aim:**

This study aimed to investigate the protective effect of *Rosmarinus officinalis* (L.) essential oil on rooster sperm motility during 4°C short-term storage.

**Materials and Methods:**

*R. officinalis* essential oil was analyzed using gas chromatography coupled to mass spectrometry to identify the active components. 10 of 45-week-old Hubbard commercial broilers were subjected to biweekly semen collections during 3 weeks. At each collection, sperm was pooled and divided into four aliquots and then diluted with Tris extender supplemented with 870, 87, or 8.7 µg/ml of *R. officinalis* essential oil, identified as treatments R, R5, and R10, respectively. Tris-based extender without any supplementation was considered as a control group. Diluted sperm was then stored at 4°C in the refrigerator and analyzed at 0, 6, 24, and 48 h using a computer-assisted sperm analyzer. Different semen parameters were measured including total motility, progressive motility, gametes velocities (straight line velocity [VSL], curvilinear velocity [VCL], and average path velocity [VAP]), amplitude of the lateral head displacement [ALH], and beat-cross frequency [BCF].

**Results:**

The phytochemical analysis of *R. officinalis* essential oil revealed the presence of 25 active components including seven major molecules: Camphor (18.88%), camphene (5.17%), 1,8-cineole (7.85%), β-thujene (13.66%), α-thujene (4.87%), chrysanthenone (12.05%), and β-cubenene (7.97%). The results showed a beneficial effect of *R. officinalis* essential oil on sperm cells motility, particularly when using the lowest concentrations, 8.7 and 87 µg/ml. Progressive motility and gametes velocities (VCL, VSL, and VAP), materializing the quality of gametes motility, showed highly statistically significant values (p<0.01) in 8.7 and 87 µg/ml treatments, especially from 6 h of storage at 4°C. Conversely, the highest concentration (870 µg/ml) showed harmful effects with a total spermicidal activity after 24 h of storage.

**Conclusion:**

The current results revealed the positive impact of *R. officinalis* essential oil on rooster sperm at 4°C short-term storage probably through fighting against oxidative stress and cold shock damages.

## Introduction

The recent years witnessed, in poultry, a growing interest in wide ranges of natural products and plant extracts, especially about their positive effects on reproduction and fertility outputs [1-3]. Essential oils, when used particularly *in vivo* as dietary supplementation, showed a remarkable potency in protecting and improving sperm parameters of several animal species [4-7]. However, when used *in vitro*, in humans as well in other animal species, essential evidenced spermicidal effects with impacts on gametes motility and plasma membrane integrity [8-12].

*Rosmarinus officinalis* is known as a rich source of polyphenols and volatile oils including molecules such as carnosic, rosmarinic acids, and camphor [13-16]. These compounds display diverse biological activities including antioxidant, anti-inflammatory, and anticarcinogenic properties. In aged roosters, Borghei-Rad *et al*. [17] reported that dietary supplementation with rosemary leaves powder improved significantly semen quality, fertility, and hatchability through fighting oxidative stress damages. Similarly, dietary supplementation using other antioxidants, including Vitamin E, selenium, Vitamin C, and enzymatic antioxidant systems, improved significantly animal health and sperm quality [18-21].

Sperm cells are known to be more vulnerable to reactive oxygen species (ROS), generated during oxidative stress, than somatic cells due to gametes cell structure and their limited contents of antioxidants. In this respect, the phospholipidic fraction of spermatozoa cell membranes [22] is the first target of ROS with negative effects on membrane fluidity and the subsequent impairment of motility, acrosome reaction, and sperm-egg interaction [23,24]. Accordingly, high membrane concentration in PUFA makes sperm cells more susceptible to lipid peroxidation, especially, during the different stages of *in vitro* storage at low temperatures compromising, thus fertility outputs [24,25].

In fact, in rooster, artificial insemination (AI) is routinely carried out after short-term storage at 4°C; however, reports evidenced that fertilizing capacity of freshly collected semen is dramatically lost after half an hour of collection [22]. Therefore, development of optimal semen extenders, including different active compounds, was considered worldwide by different research groups to protect sperm cells against motility damages. The adopted strategies consisted essentially of protecting cell membrane against cold shock and oxidative stress [26,27].

In this respect, numerous studies, using rosemary leaves extract, containing rich fractions of polyphenols, have reported positive effects on frozen semen in several species including boar [28], dog [29], and ram [30]. However, no protective effect was reported when using *R. officinalis* essential oil, and recently, Elmi *et al*. [31] observed similar motilities when comparing the control to sperm treated *in vitro* with 0.2 mg/ml of *R. officinalis* essential oil. Overall, all the reported data independently on the considered plant showed an obvious spermicidal activity.

Thus, to the best of our knowledge, no previous records reported the usefulness of essential oils as protective factors during semen preservation at low temperatures. This study aimed to investigate the interest of *R. officinalis* essential oil in the preservation of rooster sperm motility during 4°C short-term storage.

## Materials and Methods

### Ethical approval

The experiment was carried out in accordance with the guidelines laid down by the Directive 2010/63/EU of the European Parliament for Animal Ethics Committee for the use of poultry birds.

### Plant material

Aerial part (stems and leaves) of *R. officinalis* was harvested during April 2015, in the region of El-Euchre, in the Department of Bordj Bou Arreridj, located in Northeastern Algeria. The harvested plants were dried in the shade for 10 days before extraction.

Extraction of the essential oil was carried out by hydrodistillation process using a Clevenger’s type apparatus (Clevenger, 1928). Distillations were carried out by boiling aerial plant parts for 3 h, and the essential oil was kept in air-tight sealed vials and stored at 4°C until further use.

### Phytochemical identification

The identification of the active compounds of the *R. officinalis* essential oil was carried out by Agilent 2890 HP-type gas chromatography coupled to mass spectrometry (Model 6890) (GC/MS), equipped with a fused silica HP-5MS capillary column (Length: 30 m, internal diameter: 0.25 mm, and film thickness 0.25 μm). The temperature of the injector was maintained at 250°C. The carrier gas was helium (0.5 ml/min) with an injection volume of 0.2 μl, and the division ratio was adjusted to 1:20. The oven temperature was programmed from 60 (8 min) to 280°C at 2°C/min and then held isothermally at 280°C for 10 min. Analysis mode: Digitized, interface temperature: 280°C, ionization type: Electronic, filament strength: 70 eV, mass analyzer type: Quadrupole, source temperature: 230°C, and empty: 65m torr.

### Animals

All experiments were conducted in accordance with the legislation governing the ethical treatment of animals. 10 of 45-week-old Hubbard commercial broiler reproductive cocks were used during the experiment. The animals were housed in conventional individual cages under 14 h of daily illumination and fed with a standard commercial food at the rate of 155 g/day/animal.

### Sperm collection

The roosters were subjected to biweekly semen collections during 3 weeks by dorso-abdominal massage as described by Burros and Quinn [32] and individual ejaculates were gently mixed and pooled. To minimize animal stress, the collection was carried out by the same operator and under the same conditions. Necessary precautions were taken during collection to avoid contamination by cloaca fluids. Sperm concentration was calculated using a computer-assisted sperm analyzer (CASA) (Sperm class analyzer, SCA Microptic, S.L., Version 3.2.0, Barcelona, Spain).

### Sperm treatment

The pooled sperm was diluted at 1/2 ratio at room temperature (18-22°C) in Tris extender (3.028 g of hydroxymethylaminomethane, 1.25 g of fructose, 1.7 g of citric acid, 800 Ul/ml of penicillin G sodium, and 1 mg/ml of streptomycin sulfate in 100 ml of distilled water) and then split into four equal aliquots. Each aliquot was diluted with Tris extender supplemented with 870, 87, or 8.7 µg/ml of *R. officinalis* essential oil, identified as treatments R, R5, and R10, respectively (26). Tris-based extender without any supplementation was considered as a control group. All samples were then stored at 4°C and analyzed at 0, 6, 24, and 48 h. The experimentation was repeated 6 times (n=6).

### Sperm motility analysis

Various sperm motility parameters were analyzed using a CASA (Sperm class analyzer, SCA Microptic, S.L., Version 3.2.0, Barcelona, Spain). The measured parameters were: Percentage of motile spermatozoa (Mob T %), percentage of progressive spermatozoa (Prog T %), curvilinear velocity (VCL μm/s), straight line velocity (VSL μm/s), average path velocity (VAP μm/s), amplitude of the lateral head displacement (ALH μm), and frequency to which the sperm head crosses the mean trajectory (beat-cross frequency [BCF]/Hertz).

### Statistical analysis

The SAS software (SAS Institute, 2001) was used for all statistical analyses. The data were checked for normal distribution with a Shapiro–Wilk test. Treatment and time effect on each motility parameters (MobT, ProgT, VCL, VSL, VAP, ALH, and BCF) were assessed by the following general linear model:

Y_ijkl_=μ+A_i_+B_j_+(AB)_ij_+e_ijk_

Y=The studied motility parameters measured on the sperm;

μ=Mean;

A_i_=Fixed effect of the medium (i=1-4: R, R5, R10, and Tris);

B_j_=Fixed effect of time (j=1-4: 0, 6, 24, and 48 h);

(AB)_ij_=Interaction between time and medium;

e_ijk_=The residual effect.

Least-squares means and standard errors (SE) were calculated for motility parameters measurements.

Differences were considered statistically significant at p<0.05.

## Results

The chemical composition of *R. officinalis* essential oil and the retention index as well as the percentages of the various compounds are listed in Table-1. 25 compounds were identified in the essential oil without fractionation. The major constituents were camphor (18.88%), camphene (5.17%), 1,8-cineole (7.85%), β-thujene (13;66%), α-thujene (4.87%), chrysanthenone (12.05%), and β-cubenene (7.97%).

**Table-1:** Main compounds of the *R. officinalis* essential oil identified by GC/MS.

Compound	RI	%
α-Pinene	8.881	1.409
Camphene	9.734	5.171
Verbenene	10.028	0.586
Sabinene	11.185	1.374
β-Pinene	11.329	0.254
*trans* β-Ocimène	12.317	0.231
α-Terpinene	13.937	0.54
1,8-Cineole	14.978	7.850
γ-Terpinene	16.852	0.667
β-Thujene	20.404	13.662
α-Thujene	21.113	4.875
Chrysanthenone	21.797	11.548
Camphor	23.176	18.88
Carveol	23.267	0.31
Pinocarvone	24.221	0.618
α-Phellandrene	24.939	0.556
Terpineol	25.460	0.904
Chrysanthenyl acetate	31.161	1.479
α-Copaene	38.554	0.517
*cis*-Jasmone	40.357	0.491
β-Cubebene	45.311	7.976
γ-Elemene	46.145	1.719
δ-Cadinene	47.798	0.625
Spathulenol	51.090	0.158
1,3-Nonadiene	71.438	0.874

*R. officinalis=Rosmarinus officinalis*, GS–MS=Gas chromatography-mass spectrometer

### Effect of storage time and sperm treatment on sperm mobility parameters

The effects of time, sperm treatment, and their interactions sperm motility are shown in Table-2. The interaction affected all the variables significantly (p<0.001). The total and progressive motility declined rapidly at 870µg/ml corresponding to the highest concentration of *R. officinalis* essential, especially after 6 h of storage. Similar observations are noted for the kinematics parameters (VSL, VCL, VAP, ALH, and BCF).

**Table-2:** Spermatozoa mobility parameters according to the effects of storage time and sperm treatment (TRT).

Motility parameters	TRT	Storage time (ST)				SEM	p value			R^2^
	
		0	6	24	48		TRT	ST	TRT*ST	
Mob T	R	71.24^a^	14.68^b^	-	-	2.83	***	***	***	0.97
	R5	89.84^a^	92.44^a^	88.96^a^	71.43^b^	2.83				
	R10	98.12^a^	91.96^a^	92.80^a^	74.00^b^	2.83				
	Tris	92.03^a^	87.03^ab^	83.93^b^	54.04^c^	2.83				
Prog T	R	30.87^a^	1.62^b^	-	-	4.72	***	***	***	0.90
	R5	50.82^a^	68.32^b^	43.25^a^	27.31^c^	4.72				
	R10	70.30^a^	56.43^b^	63.08^ab^	36.48^c^	4.72				
	Tris	64.94^a^	39.87^b^	26.86^bc^	15.18^c^	4.72				
VSL	R	9.24^a^	4.27^b^	-	-	0.39	***	***	***	0.06
	R5	12.02^a^	9.90^b^	7.74^c^	10.96^d^	0.18				
	R10	11.79^a^	14.23^b^	10.32^c^	11.71^a^	0.23				
	Tris	13.21^a^	8.78^b^	4.22^c^	8.55^b^	0.24				
VCL	R	32.48^a^	18.17^b^	-	-	0.92	***	***	***	0.11
	R5	40.47^a^	34.60^b^	28.69^c^	39.05^d^	0.44				
	R10	43.57^a^	57.07^b^	42.46^a^	38.05^c^	0.56				
	Tris	45.90^a^	28.46^b^	28.53^b^	32.56^c^	0.62				
VAP	R	17.35^a^	8.47^b^	-	-	0.47	***	***	***	0.09
	R5	22.56^a^	20.35^b^	17.52^c^	20.24^b^	0.26				
	R10	23.11^a^	29.31^b^	22.04^c^	20.24^d^	0.30				
	Tris	24.20^a^	18.42^b^	11.00^c^	17.55^d^	0.35				
ALH	R	2.12^a^	1.76^b^	-	-	0.05	***	***	***	0.15
	R5	2.53^a^	2.23^b^	1.48^c^	2.95^d^	0.02				
	R10	3.03^a^	2.33^b^	2.55^c^	2.89^d^	0.03				
	Tris	2.74^a^	1.87^b^	1.41^c^	2.62^d^	0.03				
BCF	R	2.98^a^	1.18^b^	-	-	0.09	***	***	***	0.08
	R5	3.62^a^	3.44^b^	3.11^c^	3.41^b^	0.05				
	R10	3.77^a^	4.94^b^	3.25^c^	3.59^d^	0.05				
	Tris	4.41^a^	3.23^b^	1.62^c^	3.05^d^	0.06				

Means followed by the same letter (a,b,c) in the same line are not statistically significant different (p>0.05).

*** p<0.001; SEM=Standard error of mean, R^2^=Coefficient of determination, VSL=Straight line velocity, VCL=Straight line velocity, VAP=Average path velocity, ALH=Amplitude of the lateral head displacement, BCF=Beat-cross frequency

### Percentage of total motility

**Figure-1:**
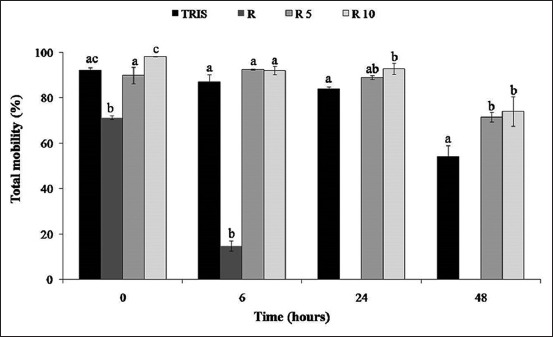
Total motility percentage of rooster spermatozoa in control (Tris) and *Rosmarinus officinalis* essential oil treatments R (870 µg/ml), R5 (87 µg/ml), and R10 (8.7 µg /ml) at 0, 6, 24, and 48 h of 4°C storage. Different letters indicate statistically significant difference (p<0.05). Values are represented as mean±standard error of the mean.

Figure-1 shows the effects of different treatments on total motility during the study period. Total motility represents the percentage of total moving spermatozoa regardless of the quality of the motility. When compared to the control group (Tris), statistically significant impact was observed in the different treatments (p<0.001). At T0, no significant difference was observed between 87 μg/ml (R5, 89.84±3.64) and 8.7 μg/ml (R10, 98.12±0.16) treatments and the control group (Tris, 92.03±1.32). The highest concentration of *R. officinalis* essential oil (R, 870 µg/ml) showed the lowest motility percentage at 0 and 6 h and a total spermicidal effect at 24 h. After 48 h of chilling storage, 87 and 8.7 µg/ml concentrations expressed the highest motility with 71.4±2.05% and 74±6.49%, respectively.

### Percentage of progressive motility

**Figure-2:**
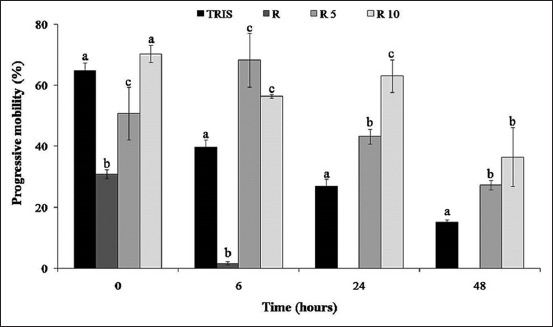
Progressive motility percentage of rooster spermatozoa in control (Tris) and *Rosmarinus officinalis* essential oil treatments R (870 µg/ml), R5 (87 µg/ml), and R10 (8.7 µg/ml) at 0, 6, 24, and 48 h of 4°C storage. Different letters indicate statistically significant difference (p<0.05). Values are represented as mean±standard error of the mean.

Progressive motility is a qualitative parameter representing gametes with forwarding motility (Figure-2). As for total motility (Figure-1), the lowest progressive gametes were observed in 870 µg/ml treatment corresponding to the highest essential oil concentration. From 6 h of storage, progressive motility was significantly preserved in 87 and 8.7 µg/ml treatments. After 24 h of storage, 87 and 8.7 µg/ml treatments showed 43.25±2.43 and 63.08±5.33% of progressive gametes compared to 26.86±2.36% of the control group (p<0.05).

### Cinematic parameters (VCL, VSL, VAP, ALH, and BCF)

**Figure-3:**
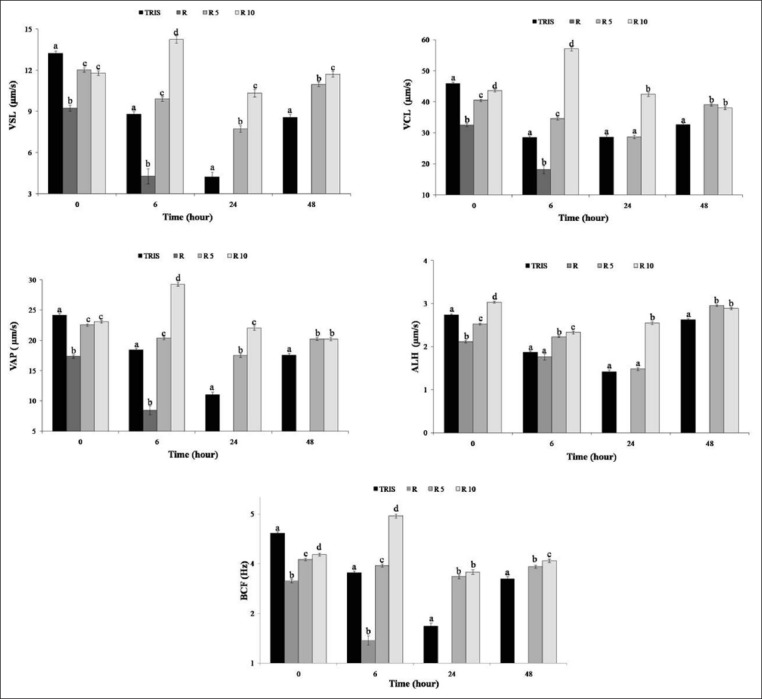
Curvilinear velocity (VCL), straight line velocity (VSL), average path velocity (VAP), amplitude of lateral movement of the head (ALH), and the beat-cross frequency (BCF/Hertz) of rooster spermatozoa in the control (Tris) and *Rosmarinus officinalis* essential oil treatments R (870 µg/ml), R5 (87 µg/ml), and R10 (8.7 µg/ml) at 0, 6, 24, and 48 h of 4°C storage. Different letters indicate statistically significant difference (p<0.05). Values are represented as mean±standard error of the mean.

Cinematic parameters including velocities (VCL, VSL, and VAP), ALH, and BCF are represented in Figure-3. The velocity of individual sperm was ranged between 0 (immotile sperm) and 100 μm/s. After 6 h of storage, highly significant differences were observed for VCL (p<0.01), VSL (p<0.01), and VAP (p<0.01) in 8.7 µg/ml treatment with 57.07±0.62, 14.23±0.26, and 29.31±0.34 μm/s, respectively. This positive impact was maintained at 24 and 48 h. Similarly, ALH and BCF showed the highest values in 87 and 8.7 µg/ml treatments.

## Discussion

Nowadays, rosemary is widely used for its antioxidant activity [33-38], antimicrobial properties [38-41], and bio-preservative in the food industry [42-44]. Recently, in poultry, Borghei-Radand *et al*. [17] reported positive effects of diet supplementation using *R. officinalis* leaves on sperm motility and fertility in aging roosters. However, very few relevant data are present regarding the protective effect of *R. officinalis* essential oil on avian spermatozoa, particularly when used *in vitro*.

In this study, the chemical composition of *R. officinalis* essential oil and the effects on rooster sperm motility during 4°C short-term were studied. Phytochemical screening of *R. officinalis* essential oil (using the GC–MS analysis) allowed the identification of 25 active compounds. According to the results, the major constituents were camphor (18.88%), 1,8-cineole (7.85%), β-thujone (13;66%), chrysanthenone (12.05%), and β-cubenene (7.97%). Similar results were reported by Zermane *et al*. [45] when investigating the chemical analysis of Algerian essential oil under optimal conditions. The analyzed essential oil presented some differences when compared to Moroccan *R*. *officinalis* essential oil. The latter showed high proportions of pinene (37-40%), cineole (58.7-63.7%), and camphor (41.7-53.8%) and *R*. *officinali*s essential oil from Tunisia is rich in cineole and contained usual monoterpenes [46,47]. Previous studies revealed that *R*. *officinalis* essential oil composition might be influenced by various factors such as the genotype of the plant, soil type, bioclimatic conditions, or the extraction method [47-49].

In the present study, the beneficial and potentially harmful effects of different concentrations of *R*. *officinalis* essential oil on rooster semen stored at 4°C for 48 h were investigated. The results demonstrated that semen quality (revealed through progressive motility and gametes velocities) was significantly preserved, especially with the lowest concentrations of the essential oil (87 µg/ml and 8.7 µg/ml). The highest dose (870 µg/ml) showed a complete spermicidal effect evidenced after 24 h of storage. This concentration caused alterations probably similar to those described in bacteria [50-52]. The mechanism described in bacteria was the ability of the essential oil to disrupt and penetrate the lipid structure of the cell wall leading to the destruction of the membranes [41,42]. Chaftar *et al*. [53] reported that *R. officinalis* essential oil was active against several strains of *Legionella pneumophila* with a concentration of <0.55 mg/mL. As well, Outaleb *et al*. [54] indicated that rosemary essential oil exhibited appreciate antibacterial activities with concentrations ranging only from 4 to 20 µg/ml. Similarly, it is reported that spermicidal activity in numerous animal species is associated with considerable damages on different spermatozoa structures including cell membranes and acrosome integrity [8-12,31,55].

According to the results of this study, the use of small concentrations *in vitro* might be beneficial to rooster spermatozoa under 4°C conditions. In fact, total and progressive motility and kinematics parameters were significantly preserved in 87 and 8.7 µg/ml treatments. After 24 h of storage, the percentage of progressive gamete was 43.25 and 63.08 at 87 and 8.7 µg/ml treatments, respectively, compared to 26.86±2.36% of the control group. Elmi *et al*. [31] reported that the total and progressive motility was significantly altered from the concentration of 0.8 mg/ml of *R. officinalis* essential oil in swine spermatozoa. This concentration was almost identical to the spermicidal concentration used in our experiment (870 µg/ml). At 0.2 mg/ml, the authors observed motility values similar to those of the control group. Conversely, when used as supplementation diet, *R. officinalis* essential oil enhanced spermatogenesis and limited lipid peroxidation in quail testicular tissue [55].

Several active molecules such as 1,8-cineole, camphor, β-thujene, chrysanthenone, β-cubebene, and camphene, known for their antioxidant activity, were identified in the phytochemical analysis (Table-1). These compounds are probably involved in the observed protective effects. These findings are in accordance with previous reports using rosemary aqueous extract (polyphenols rich fraction) in boar [28,56] and buck [57]. The beneficial effects of *R*. *officinalis* essential oil may be related to the antioxidant activity limiting lipid peroxidation and membrane damages during the chilling storage.

## Conclusion

The present work reported the usefulness of a low concentration of *R*. *officinalis* (8.7 μg/ml) in the preservation of rooster semen at 4°C for a period long enough to manage successfully AI practice. However, further studies are needed to understand the mechanisms of action and the real impact on fertility outputs.

## Authors’ Contributions

MI and LT have conceived and designed the study. LT and BA conducted the study in the laboratory. NM analyzed the data and performed statistical analysis. LT and YB drafted and revised the manuscript under the guidance of MI. All authors read and approved the final manuscript.
